# Socio-economic inequity in demand for insecticide-treated nets, in-door residual house spraying, larviciding and fogging in Sudan

**DOI:** 10.1186/1475-2875-4-62

**Published:** 2005-12-15

**Authors:** Obinna Onwujekwe, El-Fatih Mohamed Malik, Sara Hassan Mustafa, Abraham Mnzava

**Affiliations:** 1 Department of Health Administration and Management, College of Medicine, University of Nigeria, Enugu Campus, Nigeria; 2 National Malaria Administration, Khartoum, Sudan; 3 Ministry of Health, Sudan; 4 World Health Organization, Eastern Mediterranean Regional Office (EMRO), Cairo, Egypt

## Abstract

**Background:**

In order to optimally prioritize and use public and private budgets for equitable malaria vector control, there is a need to determine the level and determinants of consumer demand for different vector control tools.

**Objectives:**

To determine the demand from people of different socio-economic groups for indoor residual house-spraying (IRHS), insecticide-treated nets (ITNs), larviciding with chemicals (LWC), and space spraying/fogging (SS) and the disease control implications of the result.

**Methods:**

Ratings and levels of willingness-to-pay (WTP) for the vector control tools were determined using a random cross-sectional sample of 720 householdes drawn from two states. WTP was elicited using the bidding game. An asset-based socio-economic status (SES) index was used to explore whether WTP was related to SES of the respondents.

**Results:**

IRHS received the highest proportion of highest preferred rating (41.0%) followed by ITNs (23.1%). However, ITNs had the highest mean WTP followed by IRHS, while LWC had the least. The regression analysis showed that SES was positively and statistically significantly related to WTP across the four vector control tools and that the respondents' rating of IRHS and ITNs significantly explained their levels of WTP for the two tools.

**Conclusion:**

People were willing to pay for all the vector-control tools, but the demand for the vector control tools was related to the SES of the respondents. Hence, it is vital that there are public policies and financing mechanisms to ensure equitable provision and utilisation of vector control tools, as well as protecting the poor from cost-sharing arrangements.

## Introduction

Malaria is the major health problem in Sudan and its prevention in the country is managed through official vector control strategies, which include annual indoor residual house spraying (IRHS), larviciding with chemicals (LWC), fogging or space spraying (SS) and recently insecticide-treated nets (ITNs). Malaria accounts for about 21% of all diseases seen at outpatient departments and 32% of inpatient admissions in health facilities in the country [1]. All the vector control activities, except ITNs, are supposed to be provided free-of-charge but, in reality, because of government budget constraints, individuals, households and communities sometimes pay or contribute money to ensure that their households or immediate neighbourhoods benefit from the activities [2]. In the case of ITNs, people pay either the full or subsidized cost of the nets.

In order to maximize the limited public and private budget for malaria vector control, there is the need to determine the extent of consumer demand for different vector control tools. Basing resource-allocation decisions for vector control on consumer preferences would ensure that the strategies are effectively implemented and sustained over the long-term as the ability to sustain disease control depends very much on "listening to the people" [3].

It could be argued that only strategies that people are most willing to pay for and preferred stand a better chance of being used successfully to control and sustain malaria vector control in Sudan. This is essential, since many vector control activities require community involvement, in the presence of budget constraints, to be successfully implemented over the long-term. Some of the vector control measures such as ITNs and, to a lesser extent, IRHS, LWC and SS also require some form of community contribution to be implemented on a sustainable basis.

Hence, the determination of consumer preferences and demand for different vector control strategies becomes pertinent, when viewed against the background of community involvement as part and parcel of vector control tools and as consumers are expected to contribute some money for the financial sustainability of the delivery of the strategies. Consumer preferences should also guide resource allocation decisions so that people preferences and potential demand for the different tools are satisfied.

Willingness-to-pay (WTP), or demand elicited using the contingent valuation method (CVM) in welfare economics, is the theoretically correct method for determining demand through the value that people attach to goods and services according [4,5]. WTP is the maximum amount of money that an individual is willing to pay for goods or services. The elicited WTP is the measure of value in cost-benefit analysis (CBA) [5,6] and it can also be used to understand the determinants of the demand for goods and services.

The method is called contingent valuation because it involves respondents directly evaluating, in monetary terms, goods or services with benefits that may not be directly measurable [7]. Asking people directly has the potential to inform about the nature, depth, and economic significance of these values [8]. Some critiques have argued against the use of CVM due existence of biases. The major criticism of the validity of CVM has been that stated WTP is a poor indicator of actual WTP [9]. However, many studies have shown that the CVM can provide valid information and is now used to correctly inform healthcare policy and resource allocation decisions in both developed and developing countries [5,10,11]. It is also been increasingly used to inform malaria control decisions [5,12-14].

Since WTP is influenced by ability-to-pay [15], it is also important to examine the extent to which different socio-economic groups are willing to pay for vector control tools and hence provide information about demand responsiveness of the different vector control measures to different socio-economic status groups. This is to ensure that the final provision of the tools is equitable and that all socio-economic groups benefit equally from public expenditures and subsidies [16].

This paper presents the findings of a study to determine how different socio-economic groups prefer and demand different malaria vector control strategies. The CVM was used to determine people willingness to pay for the vector control strategies. The elicited WTP estimates were the measures of people demand for the different vector control tools. The ranking of preferences for the different vector control tools, using the rating method and the WTP were also compared, so as to determine the extent to which the results for the two methods converge, providing information on the usefulness of the simpler rating scale in understanding demand.

## Methods

### Study area and sampling

A sample size of 720 households was selected from six localities, which were drawn from Gezira and Khartoum states. The localities selected in Gezira state were Wad Medani (urban), Umra-Qura (peri-urban) and Medina Arab (rural). Similarly, Omdurman (urban), Bahry (peri-urban) and Albuga (rural) were selected in Khartoum State. The peri-urban areas are areas that surround known urban areas. Random sampling was used to decide on the households for which information was collected. The respondents in each household were either the household head or the spouse. However, in the event that these two people were not around, information was collected from an available adult household member. Where there was no available adult household member, that particular household was skipped.

### Context of current financing of the vector control strategies

#### Indoor residual house spraying (IRHS)

At national level, the government funds 60% of the cost of IRHS and the World Health Organization (WHO) funds about 20% of IRHS costs [2]. The rest of the funding for IRHS at the national level comes from private companies, public boards and the Gambiae control project in Northern Sudan. At the state level in Gezira State, the government funds 75% of IRHS costs and WHO funds the rest. In Khartoum state, the State Malaria Administration (SMA) is not involved in IRHS. Consumers contributed minimal amounts of money towards the spraying of their homes [2].

#### Insecticide-treated nets (ITNs)

The national government funds the promotion of ITNs[2]. The consumers completely financed the purchase of their ITNs in all localities. However, the price of the nets differed from district to district. In the Khartoum state localities of Bahry and Omdurman, the nets were sold at 650 dinars each, while they were sold at 600 dinars each in Albugaa, but in the Gezira state localities, the nets were sold at 750 dinars per net [2]. In Wad Medani, the Red Crescent buys nets and in Khartoum state, an Islamic organization called Zakat buys the nets, which they respectively distribute to poor people and pregnant women [2]

#### Larviciding with chemicals (LWC) and space spraying (SS)

The government funds 90% of costs of LWC, while WHO funds 10% [2]. The funding for LWC is 75% government and 25% WHO at the state level. Only the government funds SS in Khartoum state, whilst in Gezira state, the proportion of funding is 50% by the government and 50% by WHO [2]. The consumers contributed very minimal amounts of money towards LWC and SS [2].

#### Context of current delivery of the vector control strategies

The government through the SMA and malaria control staff at local level are the major deliverers of IRHS, LWC and SS [2]. There is no clear established pattern for distribution of ITNs. At the national level, the personnel of the National Malaria Administration deliver the ITNs to the states, through the SMA and the state malaria control managers then deliver the nets to the localities [2]. However, the SMA also sells the nets directly to consumers in their premises. In Bahry and Omdurman localities, the malaria control staff, volunteers and civil societies are used to sell ITNs. The government does not pay them any money, but they are allowed to retain 25 to 50 dinars per net sold as a commission [2]. Also in Umra-Qura, village committees and teachers are also used to sell the ITNs and the people are allowed to add a margin of up to 50 dinars on top of the locality price, as their commission [2].

#### Determination of levels of willingness to pay (WTP)

Pre-tested, interviewer-administered questionnaires in the local language were used to collect data from householders. The questionnaire was pre-tested in a locality in Khartoum state using 20 people and relevant corrections were effected before the data collection. The respondents were asked to rate their preferences for the vector control tools before determining WTP. The ratings used ranged from one to four, where one was the lowest (least preferred) and 4 was the highest (most preferred).

The bidding game question format was used to elicit WTP, after presenting scenarios that described the vector control tools, as well as payment vehicle which was user fees to the respondents. In the scenario, the respondents were told that the vector control tools all chase away and kill mosquitoes and hence they can reduce the number of times one gets malaria. Then they were told that their preferences as well as the maximum amounts of money that they would pay for each of the four strategies would be elicited. They were also informed that the information was needed by the government in order to decide on how to allocate funds for the prevention of malaria, in order to ensure that the best results are achieved. The respondents were allowed to ask questions before their levels of WTP were elicited.

**Figure 1:**
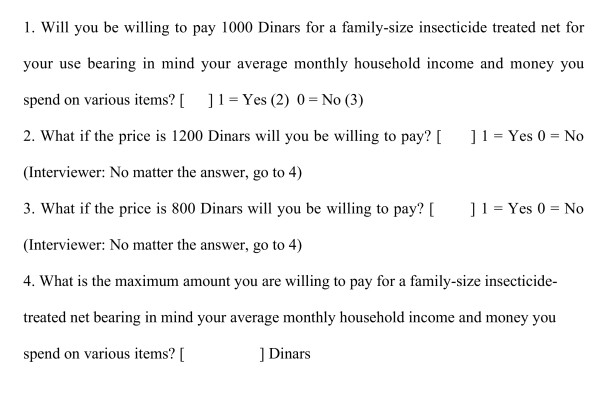
Description of the bidding game iteration using WTP for ITNs as an example.

There were four WTP iterations and the final point on the BG iteration was an open-ended question that elicited respondents' maximum WTP amount for the different tools. A uniform iteration and starting-point 1,000 Sudanese dinars (SD) were used for the four valuations, hence the study was not designed in such a way that starting-point bias could be controlled for. Starting-point bias means that the respondents are influenced by the amount used to start the bidding and may think that it represents the value of the good or service in question. Thus, the final WTP amounts they give will cluster around the starting bid. However, we did not envisage that the bias would exist in the study since there is mixed evidence about its existence [17]. The bidding game iteration using WTP for ITNs as an example is presented as figure 1.

### Data analysis

Principal components analysis (PCA) was used to generate a household SES index [18] that was used to examine socio-economic differences in WTP. Information on ownership of a radio, bicycle, television, satellite dish, refrigerator, motorcycle, motor car together with per capita weekly household value of food (expenditures plus home production) was used to generate the index. The ratio of the mean WTP of the poorest SES (Q1) over that of the least poor group (Q4) was the measure of inequity and it showed the level of gap that has to be bridged in order to improve the condition of the poorest households [18]. Tabulations, testing of means and non-parametric tests were used to decompose WTP into SES quartiles and to test whether the valuations were statistically significantly related to SES. Chi-square tests were also used to analyse whether the elicited WTP estimates differed across the four different vector control tools.

Multiple regression analyses using the Tobit model was used to test the theoretical validity of elicited WTP. Theoretical validity test in a contingent valuation study is used to assess whether hypothesized theoretical relationships between the elicited WTP and its explanatory variables are supported by the data [19]. If CVM results are valid, the estimated parameters should normally be in accordance with prior expectations [20]. A full-to-reduced modelling approach was used in order to arrive at the best regression models. The independent variables with the smallest t-statistic, and whose removal did not adversely affect the other coefficients nor the prediction of the models were removed sequentially. The F-test for the hypothesis that the coefficient of that variable is zero was used to decide whether the variable would be finally dropped or re-entered into the regression. The variables were finally dropped if the probability associated with the F-test was more than 0.10. Scatter plots of the residuals versus the predicted values were used to check for heteroscedasticity, which is a common problem with cross-sectional survey data.

Tobit model was used because there were many respondents stated zero WTP amounts. In this case, ordinary least squares (OLS) regression analysis is not an appropriate model for the econometric analysis because WTP was limited by zero values. This is because if the dependent variable is limited in some way, OLS estimates are biased [21]. OLS in this case fails to account for the qualitative differences between the limit observations (those with zero WTP) and the non-limit observations [22]. Omitting the limit observations creates bias. Ignoring them would be throwing away information, but including them as though they were ordinary observations also creates bias [21].

## Results

### Characteristics of the respondents

A total of 720 respondents were interviewed and most of them were either the head of the household or the spouse (Table 1). Also, most of the respondents were females, had some education and were married. The most common household asset holdings were television and radio sets, while satellite dishes and motorcars were the least common household asset holdings.

**Table 1:** Socio-economic and demographic characteristics of the respondents

	**n (%) N = 720**
Status of the respondent in the household	
• Household head	334 (46.9)
• Spouse	338 (46.4)
• Representative of household	48 (6.7)
Gender of the respondents	
• Female	402 (55.8)
• Male	318 (44.2)
Age in years (Standard deviation)	41.1 (12.4)
Number of household residents (Standard deviation)	6.3 (2.8)
Whether respondent had some education	
• No	155 (21.5)
• Yes	565 (78.5)
Marital status of the respondent	
• Single	78 (10.8)
• Married	642 (89.2)
Food value in Sudanese Dinars	
• Average weekly food value (S.D.)	9415.8 (17650.4)
• Average weekly per capita food value (S.D.)	1731.3 (3798.9)
Household asset ownership	
• Household owns a television set	504/720 (70%)
• Household owns a radio	506/720 (70.3%)
• Household owns a refrigerator	388/720 (53.9%)
• Household owns a satellite dish	87/633 (12.1%)
• Household owns a motorcar	106/614 (14.7%)

### Rating of preferences and WTP (demand) for the malaria control tools

IRHS received the highest proportion of highest preferred rating (41.0%) followed by ITNs (23.1%) and the preferences for LWC and space spraying were almost equal at about 18% a piece (Table 2). Majority of the respondents were willing to pay something for the vector control tools. However, using WTP to determine the respondents' demand for the vector control strategies, ITNs had the highest mean WTP followed by IRHS, while LWC had the least (Table 2). The zero WTP responses represented people that were willing to pay nothing for the interventions. The ratings and WTP were statistically significantly different across the four vector control tools.

**Table 2:** Preferences and WTP for the vector control tools

	ITNS	IRHS	LWC	SS	Chi square (p-value)
Most preferred (rating) n (%)	166 (23.1)	294 (41.0)	131 (18.2)	129 (17.9)	88.0 (0.001)
Number willing to pay n (%)	415 (57.6)	362 (50.3)	324 (45.0)	323 (44.9)	9.6 (0.02)
Mean WTP amount (Std. dev)	334.0 (763.0)	290.4 (549.0)	188.1 (524.1)	248.0 (765.6)	44.1 (0.001)

### Influence of socio-economic status (SES) on willingness to pay for control tools

The WTP results showed that as SES increases, the demand increases. However, although Q4 had the highest levels of WTP and Q1 the least in the four vector control tools respectively, WTP did not increase monotonically between Q2 and Q3 except with regards to SS. The inter-quartile ratios show that there was more inequity in WTP for IRHS and ITNs, whilst WTP for LWC was the least inequitable. The trend of increasing valuation of benefit as SES increases in the four vector control strategies were statistically significantly different (p < 0.001).

### Multiple regression analysis

The reduced tobit regression models showed that SES was positively and statistically significantly related to WTP across the four vector control tools (Table 4). The respondents' rating of IRHS and ITNs significantly explained their levels of WTP for the two tools. In addition, male respondents stated higher levels of WTP for IRHS, ITNs and SS. The other statistically significant variables were age in IRHS and it shows that older people stated lower WTP than younger people; household heads were more willing to pay for SS than their representatives; and people who had some education were more willing to pay for LWC. The regression analyses were all statistically significant (p < 0.01). There was no evidence of heteroscedasticity.

## Discussion

There were socio-economic differences in WTP (demand) for all the vector control tools, with the poorer respondents stating smaller WTP amounts than the better off-households. The positive association of WTP and ability to pay is an indicative factor of the validity of WTP or demand elicited through the contingent valuation method, as WTP is linked to ability to pay [15]. The ratings of the preferences were also very good indicators of the value that people attached to the different vector control tools.

In general, the finding that IRHS was mostly preferred by the respondents could be used to argue for the intensified implementation of the vector control tool in Sudan. However, this is tempered by the fact that the actual numbers of people that were willing to pay for ITNs and the levels of WTP were higher than that of IRHS. It was interesting to find that an almost equal number of people mostly preferred LWC and SS and stated positive WTP amounts for both of them respectively.

The study is limited by the fact that there was no collection of information on reasons that certain interventions were more preferred and this could be done in future studies using qualitative and or quantitative methods. Nonetheless, it is possible that ITNs had the highest WTP maybe because people knew that IRHS, LWC and SS were relatively free, while they had to pay for ITNs. It is also possible that they also knew the price of ITNs, which could have made them to quote higher WTP amounts for ITNs.

The socio-economic inequities that were found in all vector control tools should be used to guide resource allocation and policy decisions on the implementation of sustainable vector control. However, the finding that WTP did not increase monotonically between Q2 and Q3 except in the case of SS is a point of cautious interpretation of the equity implications of the result. Nonetheless, the finding that Q4 had the highest levels of WTP and Q1 lowest level of WTP for all vector control tools supports the presence of inequity in elicited WTP. The finding that overall mean WTP for ITNs was 334 SD and WTP for the least-poor group was 443 SD does not mean that it reflects the view of the poorest. These findings call for caution to be exercised in using WTP data for policy making without controlling for SES effect. Previous studies have shown that WTP vary across SES [23].

It is advocated that WTP data should always be decomposed by SES or other groupings of interest so as to ensure that the higher WTP of the better-off households do not unduly sway policy and resource allocations, which may result in more inequity. Also, WTP approximates the need of people for goods and services, although this is limited by level of ability to pay, which corresponds to the SES of the respondents. This implies that poor people despite needing more of vector control tools would not be willing and able to buy the quantity that they need because of their limited level of ability to pay. Hence, it would be good in future studies to also ask respondents the maximum number of commodities they would buy at their maximum stated WTP amounts, as a proxy for understanding the level of actual need for different interventions.

The fact that different starting-points were not used in the bidding game iteration could have led to possible starting-point bias in the elicited WTP. However, if the respondent's bid reflect his or her "true" value of the good or service, then it should not matter what initial amount (or starting point) the enumerator uses to begin the bidding game [24]. Also, in real markets with differential pricing for the same good, people may still buy the goods in places where the prices are highest and this is not a reflection of any bias. Hence, considering the fact that the elicited WTP were theoretically valid, the possibility of starting-point bias weakening the results is limited.

The other determinants of WTP apart from SES from the Tobit analysis could also be used to guide how to ensure sustainable financing and provision of the vector control tools. Expectedly, males were more willing to pay for ITNs and IRHS and this is a confirmation of previous studies in healthcare that also found similar phenomenon [14,25]. This is because men are more financially empowered than females. Hence, females, especially those at risk of malaria such as pregnant women, should be specially protected either using free or subsidized nets, as is being done in Tanzania and in other malaria endemic areas [26,27]. This intervention could reduce maternal morbidity and mortality due to malaria, and help the country in attaining the millennium development goal of reducing maternal mortality and morbidity. The results also imply that if people are more aware of the LWC, they will demand it more, from the implication of the positive association of education and valuation of benefit of LWC.

## Conclusion

The relative ranking of preferences for the vector control tools differed depending on whether the rating scale of WTP was used. Hence, it could be argued that the level of convergence of both methods was not very strong. However, an explanation for the finding, which is mostly due to IRHS having the highest rating and ITNs the highest WTP could be because the respondents reasoned that they had to pay the full cost of ITNs. Conversely, their current experience with IRHS is that the tool could still be delivered on payment of partial costs.

It is possible that people's previous personal experiences as well as the nature of the vector control tools influenced their preferences and demand. The vector control tools, which arguably confer private benefits and are not strictly public goods were more highly preferred and demanded than LWC and SS, which are strictly public goods and do not confer private benefits. It is possible that because people knew that LWC and SS would be provided, whether or not they paid, and that even if they paid, nobody would be excluded from benefiting, the preferences and WTP for these two tools were lower than for ITNs and IRHS. Thus, one could argue that there was free-riding in stating their WTP for LWC and SS and also possibly IRHS. Free-riding is a bias that has been documented in eliciting WTP for public goods [19]. Approaches for limiting such strategic bias in eliciting WTP would include using more context-specific question formats to elicit WTP and participatory methods to develop the scenarios [5].

The paper shows that there is high level of WTP (demand) for the malaria vector control, although the WTP depended on SES. Hence, if resource allocations and decisions on optimal levels of user fees are made using the overall mean WTP as a guide, without controlling for SES effects, the poorest groups would not benefit, whilst the least poor group would benefit and still have a consumer surplus. Hence, it is vital that there are public policies and financing mechanisms to protect the poor from cost-sharing arrangements and to ensure that they derive equal benefit from the use of all the vector control tools. In the case of IRHS and ITNs, vouchers [26,27], subsidies and fee exemptions should be used to ensure that there is equity in the use of ITNs and IRHS.

**Table 3:** Socio-economic differentials of WTP for vector control tools

	WTP for IRHS Mean (SD)	WTP for ITNs Mean (SD)	WTP for LWC Mean (SD)	WTP for SS Mean (SD)
Quartile 1	171.5 (332.7)	171.9 (335.4)	149.5 (772.6)	168.2 (780.4)
Quartile 2	286.4 (480.1)	389.9 (832.7)	182.5 (423.0)	203.8 (448.5)
Quartile 3	259.8 (540.9)	317.4 (835.35)	135.6 (297.8)	231.9 (758.6)
Quartile 4	443.3 (734.5)	455.3 (889.1)	283.7 (472.0)	388.8 (973.8)
Chi-square (p-value)	37.4 (.0001)	34.1 (.0001)	40.8 (.0001)	28.1 (.0001)
Q1:Q4 ratio	0.39	0.38	0.53	0.43

**Table 4:** Reduced Tobit models for determining the factors that explain WTP for four vector control tools

	IRHS Coefficient (SE)	ITNs Coefficient (SE)	LWC Coefficient (SE)	SS Coefficient (SE)
Status in household	--------------	85.5 (82.0)	72.95 (45.43)	149.6 (87.0)*
Number of residents	--------------	-------------	---------------	--------------
Sex	199.3 (66.5)***	310.7 (122.8)**	---------------	245.7 (133.7)*
Age	-6.4 (2.6)**	-5.47 (4.36)	---------------	-5.7 (4.8)
School	-------------	-------------	176.5 (99.5)*	-------------
Marital status	--------------	213.0 (198.8)	---------------	251.7 (212.5)
Rating of vector control tool	100.4 (28.5)***	187.9 (42.1)***	---------------	71.7 (49.0)
SES index	158.5 (21.8)***	162.4 (34.1)***	105.8 (26.7)***	177.3 (37.2)***
Constant	-93.1 (135.8)	-784.7 (260.9)***	-457.1 (93.1)***	-774.0 (298.4)**
LR Chi2	67.52	58.68	27.29	36.19
P-value	0.0001	0.0001	0.0001	0.0001

Note: p < 0.10 = *; p < 0.05 = **; p < 0.01 = ***

## Authors' contributions

OO and AM conceived and designed the study. OO, EM and SHM participated in data collection. All the authors participated in data analysis. OO wrote the first draft and all the authors revised the drafts until the final draft was produced for publication.
